# Severe Periodontitis Is Inversely Associated with Coffee Consumption in the Maintenance Phase of Periodontal Treatment

**DOI:** 10.3390/nu6104476

**Published:** 2014-10-21

**Authors:** Tatsuya Machida, Takaaki Tomofuji, Daisuke Ekuni, Tetsuji Azuma, Noriko Takeuchi, Takayuki Maruyama, Shinsuke Mizutani, Kota Kataoka, Yuya Kawabata, Manabu Morita

**Affiliations:** Department of Preventive Dentistry, Okayama University Graduate School of Medicine, Dentistry and Pharmaceutical Sciences, 2-5-1 Shikata-cho, Kita-ku, Okayama 700-8558, Japan; E-Mails: tomofu@md.okayama-u.ac.jp (T.T.); dekuni7@md.okayama-u.ac.jp (D.E.); tetsuji@md.okayama-u.ac.jp (T.A.); takeuti@md.okayama-u.ac.jp (N.T.); t-maru@md.okayama-u.ac.jp (T.M.); pbme8eie@s.okayama-u.ac.jp (S.M.); de18017@s.okayama-u.ac.jp (K.K.); de18019@s.okayama-u.ac.jp (Y.K.); mmorita@md.okayama-u.ac.jp (M.M.)

**Keywords:** periodontitis, coffee, nutritional sciences, cross-sectional study

## Abstract

This cross-sectional study addressed the relationship between coffee consumption and periodontitis in patients during the maintenance phase of periodontal treatment. A total of 414 periodontitis patients in the maintenance phase of periodontal treatment completed a questionnaire including items related to coffee intake and underwent periodontal examination. Logistic regression analysis showed that presence of moderate/severe periodontitis was correlated with presence of hypertension (Odds Ratio (OR) = 1.99, *p* < 0.05), smoking (former, OR = 5.63, *p* < 0.01; current, OR = 6.81, *p* = 0.076), number of teeth present (OR = 0.89, *p* < 0.001), plaque control record ≥20% (OR = 1.88, *p* < 0.05), and duration of maintenance phase (OR = 1.07, *p* < 0.01). On the other hand, presence of severe periodontitis was correlated with smoking (former, OR = 1.35, *p* = 0.501; current, OR = 3.98, *p* < 0.05), coffee consumption (≥1 cup/day, OR = 0.55, *p* < 0.05), number of teeth present (OR = 0.95, *p* < 0.05), and bleeding on probing ≥ 20% (OR = 3.67, *p* < 0.001). There appears to be an inverse association between coffee consumption (≥1 cup/day) and prevalence of severe periodontitis in the maintenance phase of periodontal treatment.

## 1. Introduction

Periodontitis is a chronic oral disease affecting all populations all over the world [1,2]. It is characterized by inflammation of the gingiva and/or destruction of the connective tissue and alveolar bone that support the teeth. Subgingival microorganisms that adhere to and grow in the periodontal pocket, along with excessive and aggressive immune response against these microorganisms, are considered to cause periodontitis. Therefore, the primary purpose of periodontal treatment is to control subgingival microorganisms.

In addition to removal of the etiological agent of periodontitis, the control of risk factors of periodontitis is also essential to maintain periodontal health. Several studies have established that factors such as tobacco use [3,4], excessive alcohol consumption [5,6], diabetes mellitus [7], and dyslipidemia [8] are risks for periodontitis. Other studies have also suggested that nutritional modulation, which is influenced by lifestyle, environmental and genetic exposure, may be involved in periodontitis [9,10]. For instance, an epidemiological study suggested a modest inverse association between the intake of green tea and periodontitis [11]. Also, recent research has reported that a high intake of fruits and vegetables may be inversely associated with periodontitis progression [12]. Furthermore, it is reported that higher daily intakes of milk and fermented foods may be protective against periodontitis [13]. However, the association between nutritional factors and periodontitis is still not completely understood.

Coffee is one of the most consumed drinks in the world. Several studies have suggested that coffee polyphenols including chlorogenic acid are potent chemopreventive agents [14,15]. Epidemiological studies demonstrate that a higher intake of coffee is associated with lower grade of nonalcoholic fatty liver disease [16], metabolic syndrome [17], liver cancer [18], and lower prevalence of oral, pharyngeal, and esophageal cancers [19]. Moreover, coffee consumption was reported to be inversely associated with markers of inflammation and endothelial dysfunction [20]. Therefore, it is possible that coffee consumption affects periodontitis.

The maintenance phase of periodontal treatment is important for maintaining the periodontal condition after initial preparation therapy, periodontal surgical therapy or therapy for recovery of oral function. It is necessary to determine the factors associated with periodontitis to maintain the periodontal condition, even in the maintenance phase of periodontal treatment. It has been shown that higher coffee consumption was associated with a significant reduction in the number of teeth with periodontal bone loss in men [21]. However, it remains unclear whether coffee consumption modulates periodontal condition during the maintenance phase of periodontal treatment.

In the present study, we hypothesized that habitual coffee consumption was associated with periodontal condition during the maintenance phase of periodontal treatment. Therefore, the purpose of this cross-sectional study was to investigate the relationship between coffee consumption and periodontal condition in the maintenance phase of periodontal treatment.

## 2. Methods

### 2.1. Study Population

Four hundred and thirty chronic periodontitis patients (mean ± standard deviation (SD), 66.4 ± 9.9 years) were recruited at the Department of Preventive Dentistry, Okayama University Hospital from June 2013 to December 2013. Chronic periodontitis was defined as ≥1 tooth sites with probing pocket depth (PPD) ≥4 mm [22]. The entrance criteria of maintenance therapy were absence of acute inflammation and no use of antibiotics for over 6 months after initial preparation.

All participants received comprehensive dental care that included non-surgical periodontal therapy consisting of oral examination, oral hygiene instructions, supra/sub-gingival debridement and scaling and root-planing of all pockets (≥4 mm) every 3 to 4 months. At the onset of the study period, they had already entered the maintenance phase for over 1 year. Period of maintenance phase (mean ± SD) was 10.9 ± 6.7 years. Exclusion criteria were pregnancy and use of antibiotic drugs within 3 months; 16 participants were excluded based on these criteria. Data of 414 participants were therefore analyzed. This study was conducted according to the guidelines laid down in the Declaration of Helsinki and all procedures involving human participants were approved by the Ethics Committee of Okayama University. After obtaining written informed consent, a detailed medical questionnaire was completed by the dentists and participants who fulfilled the study requirements were enrolled.

### 2.2. Oral Examination

PPD and clinical attachment level (CAL) were determined at six sites (mesio-buccal, mid-buccal, disto-buccal, mesio-lingual, mid-lingual and disto-lingual) on all teeth using a color-coded probe (Hu-Friedy, Chicago, IL, USA). Sites that bled upon gentle probing with 25 g of probing force were recorded, and the percentage of sites with bleeding on probing (BOP) *versus* total sites was measured in each participant. Plaque levels (plaque control record (PCR)) were measured after staining with erythrosine, and were recorded in terms of presence or absence adjacent to the gingival margin at four sites (mesial, distal, buccal and lingual) around each tooth [23]. All clinical procedures were performed by six trained and calibrated dentists (Takaaki Tomofuji, Daisuke Ekuni, Tetsuji Azuma, Noriko Takeuchi, Takayuki Maruyama, and Tatsuya Machida). In order to check the intra- and inter-examiner agreement, measurements of PPD and CAL were recorded and repeated within a 2-week interval in eight randomly selected chronic periodontitis patients. Data were analyzed with the non-parametric κ test and intra-class correlation was determined. The κ coefficients for intra- and inter-examiner and intra-class correlation coefficients were >0.8.

### 2.3. Physical Assessment

The weight and height of participants were recorded from the questionnaire. Body mass index (BMI) (weight in kilograms per height in meter^2^) was calculated for each participant.

### 2.4. Questionnaire

Dietary intake was assessed by a self-administered food frequency questionnaire. The questionnaire provided four categories of response to describe participants’ frequency of vegetable and fruit consumption: ≤2 times/month; 1 to 2 times/week; 3 to 4 times/week; or every day [24]. We combined the categories of vegetable and fruit consumption into two categories: ≤3 to 4 times/week and every day. The questionnaire provided three categories of responses to describe participants’ green tea and coffee consumption frequency: <1 cup/day; 1 to 3 cups/day; and ≥4 cups/day [19]. We further combined the categories of coffee consumption and green tea consumption into two categories: <1 cup/day and ≥1 cup/day [11,25]. The volume of a typical cup of green tea and coffee was 150 mL. The questionnaire also included details of the coffee typically consumed, details of alcohol drinking (never, former, or current) and smoking (never, former, or current), exercise habits (2 categories divided by median (120 min/week)), and tooth brushing frequency.

We also collected data via questionnaire twice at an interval of more than 1 month from the present participants (*n* = 48) and performed Spearman’s rank coefficient in test-retest method. In addition, we collected data in two different seasons to assess the seasonal changes in coffee, green tea, vegetable, and fruits consumption (*n* = 15).

### 2.5. Assessment of Periodontitis Severity

Periodontitis severity was determined using the consensus definitions published by the joint Center for Disease Control/American Association of Periodontology (CDC/AAP) working group [26]. Severe periodontitis was defined as ‘≥2 interproximal sites with CAL ≥6 mm (not on same tooth) and ≥1 interproximal site with PPD ≥5 mm’. Moderate periodontitis was defined as ‘≥2 interproximal sites with CAL ≥4 mm (not on same tooth), or ≥2 interproximal sites with PPD ≥5 mm (not on same tooth)’. Mild periodontitis was defined as ‘≥2 interproximal sites with CAL ≥3 mm (not on same tooth), and ≥2 interproximal sites with PPD ≥4 mm (not on same tooth) or one site with PPD ≥5 mm’. No periodontitis was defined as ‘no evidence of mild, moderate, or severe periodontitis’. These criteria were applied to all permanent teeth except for third molars.

### 2.6. Confounding Factors Definition

The following variables were considered to be confounding factors: gender, age (<65 years, ≥65 years), BMI (<18.5 kg/m^2^, 18.5 to 22.9 kg/m^2^, ≥23.0 kg/m^2^) [27], diabetes mellitus, hypertension, heart disease, dyslipidemia, smoking (never, former, or current), alcohol consumption (never/former or current), vegetable and fruit consumption (≤3–4 times/week or every day) [24], coffee consumption (<1 cup/day, ≥1 cup/day) [25] and green tea consumption (<1 cup/day, ≥1 cup/day) [11], exercise habits (<120 min/week, ≥120 min/week), number of teeth present, BOP (%) (≥20, <20) [28], PCR (%) (≥20, <20) [29], and duration of maintenance phase.

### 2.7. Statistical Analysis

Chi-square test or unpaired *t*-test were used to explore potential confounders for periodontitis severity [30,31,32]. Logistic regression analyses were performed with moderate/severe periodontitis and severe periodontitis as dependent variables. Independent variables were selected when the *P* value was <0.20 for the chi-square test or unpaired *t*-test in each variable and based on previous studies because it has been suggested that potential confounders should be eliminated only if *P* > 0.20 in order to prevent residual confounding [33]. The significance level was two-sided for each statistical comparison. Reported *P* values in logistic regression analyses were considered statistically significant if less than 0.05. We assessed the model fit using Hosmer and Lemeshow test.

Analyses were performed using a statistical package (IBM SPSS statistics version 20, IBM Japan, Tokyo, Japan).

## 3. Results

The Spearman’s rank coefficients in the test-retest method of all variables were more than 0.8. In addition, concordance rates of frequency for coffee, green tea, vegetable, and fruits consumption in different seasons were 0.722, 0.431, 0.452, and 0.185, respectively. Table 1 presents the characteristics of the participants. The prevalence of never alcohol drinker, vegetable consumption every day, green tea consumption ≥1 cup/day was more than 60% and that of coffee consumption ≥1 cup/day was more than 50%. On the other hand, the prevalence of the participants with BOP ≥20% was less than 15%. All of the participants’ brushing frequency were ≥2 times/day.

**Table 1:** Characteristics of the participants (*n* = 414).

Variables	Categories	*n* (%) or mean ± SD
Gender	Male	86 (20.8)
	Female	328 (79.2)
Age (years)	<65	154 (37.2)
	≥65	260 (62.8)
Presence of diabetes mellitus	Yes	34 (8.2)
Presence of hypertension	Yes	101 (24.4)
Presence of heart disease	Yes	30 (7.2)
Presence of dyslipidemia	Yes	64 (15.5)
Smoking	Never	366 (88.4)
	Former	35 (8.5)
	Current	13 (3.1)
Alcohol consumption	Never	278 (67.1)
	Former	15 (3.6)
	Current	121 (29.2)
Vegetable consumption	<2 times/month	3 (0.7)
	1–2 times/week	34 (8.2)
	3–4 times/week	105 (25.4)
	every day	272 (65.7)
Fruit consumption	<2 times/month	19 (4.6)
	1–2 times/week	78 (18.8)
	3–4 times/week	92 (22.2)
	every day	225 (54.3)
Green tea consumption	<1 cup/day	130 (31.4)	
	1–3 cups/day	164 (39.6)	
	≥4 cups/day	120 (29.0)	
Coffee consumption	<1 cup/day	170 (41.1)	
	1–3 cups/day	208 (50.2)	
	≥4 cups/day	36 (8.7)	
Exercise habits	<120 min/week	197 (47.6)	
	≥120 min/week	217 (52.4)	
BMI	<18.5	49 (11.8)	
	18.5 to 22.9	215 (51.9)	
	≥23.0	150 (36.2)	
Number of teeth present		23.4 ± 5.3	
PPD (mm)		1.97 ± 0.4	
CAL (mm)		2.58 ± 0.9	
BOP (%)	≥20	58 (14.0)	
PCR (%)	≥20	170 (41.1)	
Severity of periodontitis	No	109 (26.3)	
	Mild	12 (2.9)	
	Moderate	204 (49.3)	
	Severe	89 (21.5)	

*n*, number; SD, standard deviation; BMI, body mass index; PPD, probing pocket depth; CAL, clinical attachment level; BOP, bleeding on probing; PCR, plaque control record.

Results of the comparisons of the participants with different periodontitis severity are shown in Table 2. Variables with *p* values less than 0.2 for the chi-square test or unpaired *t*-test comparing the participants who had no/mild periodontitis with the participants who had moderate/severe periodontitis were gender, age, BMI, hypertension, smoking, number of teeth present, PCR, and duration of maintenance phase. Similarly, variables with *p* values less than 0.2 for the chi-square test or unpaired *t*-test comparing the participants who had no/mild/moderate periodontitis with the participants who had severe periodontitis were age, hypertension, smoking, alcohol consumption, vegetable consumption, coffee consumption, feature of drinking coffee (consumed with sugar), number of teeth present, and BOP.

Table 3 represents the results of the logistic regression analysis with moderate/severe periodontitis as dependent variable. Moderate/severe periodontitis was related with presence of hypertension (Odds ratio (OR) = 1.99, *p* < 0.05), smoking (former, OR = 5.63, *p* < 0.01; current, OR = 6.81, *p* = 0.076), number of teeth present (OR = 0.89, *p* < 0.001), PCR ≥20% (OR = 1.88, *p* < 0.05), and duration of maintenance phase (OR = 1.07, *p* < 0.01) after adjusting for gender, age, BMI, hypertension, smoking, number of teeth present, PCR, and duration of maintenance phase.

**Table 2:** Comparisons of the variables of the participants with severity of periodontitis.

Variables	Categories	Severity of Periodontitis		*P* Value *	Severity of Periodontitis		*P* Value *
		No/Mild	Moderate/Severe		No/Mild/Moderate	Severe	
		(*n* = 121)	(*n* = 293)		(*n* = 325)	(*n* = 89)	
		*n* (%) or mean ± SD	*n* (%) or mean ± SD		*n* (%) or mean ± SD	*n* (%) or mean ± SD	
Gender	Male	18 (14.9)	68 (23.2)	0.057	64 (19.7)	22 (24.7)	0.300
Age, years	≥65	59 (48.8)	201 (68.6)	<0.001	197 (60.6)	63 (70.8)	0.079
BMI	<18.5	18 (14.9)	31 (10.6)	0.108	37 (11.4)	12 (13.5)	0.457
	18.5 to 22.9	68 (56.2)	147 (50.2)		174 (53.5)	41 (46.1)	
	≥23.0	35 (28.9)	115 (39.2)		114 (35.1)	36 (40.4)	
Presence of diabetes mellitus	Yes	8 (6.6)	26 (8.9)	0.446	24 (7.4)	10 (11.2)	0.241
Presence of hypertension	Yes	17 (14.0)	84 (28.7)	0.002	72 (22.2)	29 (32.6)	0.042
Presence of heart disease	Yes	9 (7.4)	21 (7.2)	0.923	23 (7.1)	7 (7.9)	0.799
Presence of dyslipidemia	Yes	19 (15.7)	45 (15.4)	0.930	52 (16.0)	12 (13.5)	0.561
Smoking	Never	117 (96.7)	249 (85.0)	0.003	293 (90.2)	73 (82.0)	0.044
	Former	3 (2.5)	32 (10.9)		25 (7.7)	10 (11.2)	
	Current	1 (0.8)	12 (4.1)		7 (2.2)	6 (6.7)	
Alcohol consumption	Never/former	86 (71.1)	207 (70.6)	0.931	235 (72.3)	58 (65.2)	0.189
	Current	35 (28.9)	86 (29.4)		90 (27.7)	31 (34.8)	
Vegetable consumption	Every day	81 (66.9)	191 (65.2)	0.732	223 (68.6)	49 (55.1)	0.017
Fruit consumption	Every day	64 (52.9)	161 (54.9)	0.702	178 (54.8)	47 (52.8)	0.742
Green tea consumption	≥1 cup/day	85 (70.2)	199 (67.9)	0.642	223 (68.6)	61 (68.5)	0.989
Coffee consumption	≥1 cup/day	75 (62.0)	169 (57.7)	0.418	202 (62.2)	42 (47.2)	0.011
Feature of drinking coffee							
Consumed with sugar ^†^	Yes	27 (23.9)	74 (27.5)	0.465	73 (24.0)	28 (35.9)	0.034
	No	86 (76.1)	195 (72.5)		231 (76.0)	50 (64.1)	
Consumed with milk ^†^	Yes	67 (59.3)	163 (61.0)	0.749	182 (60.1)	48 (62.3)	0.716
	No	46 (40.7)	104 (39.0)		121 (39.9)	29 (37.7)	
Decaffeinated ^†^	Yes	11 (10.7)	21 (8.8)	0.581	25 (9.1)	7 (10.3)	0.767
	No	92 (89.3)	218 (91.2)		249 (90.9)	61 (89.7)	
Using of coffee filter ^†^	Yes	58 (54.7)	135 (53.4)	0.814	156 (54.5)	37 (50.7)	0.555
	No	48 (45.3)	118 (46.6)		130 (45.5)	36 (49.3)	
Exercise habits	≥120 min/week	62 (51.2)	155 (52.9)	0.758	174 (53.5)	43 (48.3)	0.382
Number of teeth present		25.3 ± 4.9	22.6 ± 5.2	<0.001	23.8 ± 5.4	21.7 ± 4.3	<0.001
BOP (%)	≥20	13 (10.7)	45 (15.4)	0.219	31 (9.5)	27 (30.3)	<0.001
PCR (%)	≥20	38 (31.4)	132 (45.1)	0.010	131 (40.3)	39 (43.8)	0.551
Duration of maintenance phase		9.3 ± 6.2	11.6 ± 6.8	0.002	10.7 ± 6.8	11.5 ± 6.4	0.370

*n*, number; SD, standard deviation; BMI, body mass index; BOP, bleeding on probing; PCR, plaque control record. Independent variables with *P* value less than 0.20 for the chi-square test or unpaired *t*-test were considered as potential confounders for periodontitis severity. * Chi-square test or unpaired *t*-test; ^†^ In the chi-square test, we excluded the participants who answered unknown/others in each variable.

**Table 3:** Logistic regression analysis with moderate/severe periodontitis as dependent variable.

Variables *	Categories	ORcrd	95%CI	*P* value ^†^	ORadj ^‡^	95%CI	*P* value ^†^
Gender	Male	1			1		
	Female	0.58	0.33, 1.02	0.059	0.81	0.42, 1.56	0.520
Age, years	<65	1			1		
	≥65	2.30	1.49, 3.54	<0.001	1.59	0.96, 2.64	0.070
BMI	<18.5	1			1		
	18.5 to 22.9	1.26	0.66, 2.40	0.492	1.19	0.58, 2.48	0.634
	≥23.0	1.91	0.95, 3.82	0.068	1.60	0.73, 3.50	0.245
Presence of hypertension	No	1			1		
	Yes	2.46	1.39, 4.36	0.002	1.99	1.07, 3.71	0.030
Smoking	Never	1			1		
	Former	5.01	1.50, 16.70	0.009	5.63	1.56, 20.32	0.008
	Current	5.64	0.73, 43.88	0.098	6.81	0.82, 56.74	0.076
Number of teeth present		0.89	0.84, 0.93	<0.001	0.89	0.85, 0.95	<0.001
PCR (%)	<20	1			1		
	≥20	1.79	1.15, 2.80	0.011	1.88	1.15, 3.07	0.011
Duration of maintenance phase		1.06	1.02, 1.10	0.002	1.07	1.03, 1.11	0.001

ORcrd, unadjusted odds ratio; ORadj, adjusted odds ratio; CI, confidence interval; BMI, body mass index; PCR, plaque control record. *P* values were considered statistically significant if less than 0.05. * Variables were selected according to *P* value less than 0.20 with the chi-square test or unpaired *t*-test in Table 2 and considering previous studies; ^†^ Wald test; ^‡^ Odds ratio adjusted by gender, age, BMI, hypertension, smoking, number of teeth present, PCR, and duration of maintenance phase. According to the Hosmer and Lemeshow test, chi-square statistic was 5.424 (*p* = 0.711).

Table 4 represents the results of the logistic regression analysis with severe periodontitis as dependent variable. Severe periodontitis was related with smoking (former, OR = 1.35, *p* = 0.501; current, OR = 3.98, *p* < 0.05), coffee consumption (≥1 cup/day, OR = 0.55, *p* < 0.05), number of teeth present (OR = 0.95, *p* < 0.05), and BOP ≥ 20% (OR = 3.67, *p* < 0.001) after adjusting for age, hypertension, smoking, alcohol consumption, vegetable consumption, coffee consumption, feature of drinking coffee (consumed with sugar), number of teeth present, and BOP.

**Table 4:** Logistic regression analysis with severe periodontitis as dependent variable.

Variables ^*^	Categories	ORcrd	95%CI	*P* value ^†^	ORadj ^‡^	95%CI	*P* value ^†^
Age, years	<65	1			1		
	≥65	1.57	0.95, 2.62	0.080	1.45	0.80, 2.62	0.218
Presence of hypertension	No	1			1		
	Yes	1.70	1.02, 2.84	0.044	1.46	0.82, 2.59	0.196
Smoking	Never	1			1		
	Former	1.61	0.74, 3.49	0.232	1.35	0.57, 3.19	0.501
	Current	3.44	1.12, 10.55	0.031	3.98	1.11, 14.29	0.034
Alcohol consumption	Never/former	1			1		
	Current	1.40	0.85, 2.30	0.175	1.45	0.82, 2.57	0.202
Vegetable consumption	≤3–4 times/week	1			1		
	Every day	0.56	0.35, 0.90	0.018	0.59	0.34, 1.00	0.050
Coffee consumption	<1 cup/day	1			1		
	≥1 cup/day	0.54	0.34, 0.87	0.012	0.55	0.32, 0.92	0.023
Coffee consumed with sugar	Yes	1			1		
	No/others/unknown	0.63	0.38, 1.06	0.081	0.69	0.39, 1.22	0.200
Number of teeth present		0.93	0.89, 0.97	0.001	0.95	0.90, 0.99	0.027
BOP (%)	<20	1			1		
	≥20	4.13	2.30, 7.41	<0.001	3.67	1.96, 6.86	<0.001

ORcrd, unadjusted odds ratio; ORadj, adjusted odds ratio; CI, confidence interval; BOP, bleeding on probing. *P* values were considered statistically significant if less than 0.05. * Variables were selected according to *P* value less than 0.20 with the chi-square test or unpaired *t*-test in Table 2 and considering previous studies; ^†^ Wald test; ^‡^ Odds ratio adjusted by age, hypertension, smoking, alcohol consumption, vegetable consumption, coffee consumption, coffee consumed with sugar, number of teeth present, and BOP. According to the Hosmer and Lemeshow test, chi-square statistic was 6.599 (*p* = 0.580).

## 4. Discussion

This cross-sectional study assessed the relationship between habitual coffee consumption and periodontal condition in the maintenance phase of periodontal treatment. We found that the group drinking ≥1 cup of coffee/day had lower prevalence of severe periodontitis than the group drinking <1 cup of coffee/day after adjusting for the independent variables. This indicates that habitual coffee consumption was related to severe periodontitis. On the other hand, there was no significant difference between the group drinking ≥1 cup of coffee/day and <1 cup of coffee/day in the prevalence of moderate periodontitis. Although habitual coffee consumption could prevent the progression of periodontitis, it may have little effect in the early stage of periodontitis.

Coffee contains some chemical compounds; the phenolic compounds of coffee (chlorogenic acid, ferulic acid, and p-coumaric acid) are known to have a strong protective antioxidant property [20]. A previous study reported that dihydrocaffeic acid, which is detected in human plasma following coffee ingestion, scavenges intracellular reactive oxygen species (ROS) [34]. These findings suggest that the systemic increase in anti-oxidative property following coffee consumption contributes to a decrease in ROS-induced damage at the local level. ROS is involved in the pathology of periodontitis [35]. Therefore, the anti-oxidative property of coffee may correlate with severe periodontitis in our findings. However, further studies are needed to clarify this point.

In this study, we divided coffee consumption status into <1 cup/day and ≥1 cup/day. A previous study [25] reported that coffee (≥1 cup/day) consumption reduced the risk of cardiovascular disease because of its antioxidant activities. Consumption of coffee (≥1 cup/day) was associated with lower risk of upper gastrointestinal cancer in a Japanese population [19]. Therefore, we estimated that consumption of ≥1 cup of coffee/day could affect the periodontitis severity because of its antioxidative properties.

In our population, the concordance rate of green tea consumption in different seasons was moderate (*n* = 15). This indicates that frequency of green tea consumption varied according to the seasons. It is reported an inverse correlation between green tea consumption and periodontitis [11]. However, since the present investigation was performed in seasons different from the previous study, similar relationship might not be observed.

All participants in this study had already received supportive periodontal care for over 1 year. Moreover, all of the participants’ brushing frequency was ≥2 times/day and the low percentage of sites with BOP reflected the periodontal status of the well-maintained patient population. This suggests that the lower prevalence of severe periodontitis in the present population was associated with coffee consumption, even when local disease activity of periodontitis was little. Although the main purpose of periodontal treatment is to eliminate subgingival microorganisms, systemic therapeutic approaches including dietary consultation may also offer clinical benefits in the prevention of severe periodontitis in patients during the maintenance phase of periodontal treatment.

Investigators have studied the association between coffee consumption and inflammation. High caffeinated coffee consumption was associated with lower plasma inflammatory parameters including E-selectin and C-reactive protein (CRP) in women with type 2 diabetes [20]. It is also known that the serum CRP concentration is progressively lower with higher intake of coffee in men with high serum γ-glutamyltransferase [36]. On the other hand, a cross-sectional study found that coffee consumption showed significant positive associations with adiponectin and total and low-density lipoprotein cholesterol, and inverse associations with leptin, high sensitivity CRP, triglycerides and liver enzymes [37]. Furthermore, a 30-year longitudinal study conducted between 1968 and 1998 [21] reported that coffee consumption might be protective against periodontal bone loss in adult men. These findings are consistent with the present concept that coffee consumption was related to severe periodontitis in the maintenance phase of periodontal treatment.

In this study, 58.9% of participants drank ≥1 cup of coffee/day. According to a previous study [24], among 38,701 participants (18,867 men and 19,834 women, aged 40–64 years) who participated in a large-scale prospective cohort study in Japan, 45.5% of participants drank ≥1 cup of coffee/day. In addition, prevalence of the participants with mild, moderate, and severe periodontitis in this study was 2.9%, 49.3%, and 21.5%, respectively. The prevalence of severe periodontitis in this study may be high, because it is reported that the prevalence of the participants with mild, moderate, severe periodontitis in the non-institutionalized United States population aged ≥65 years were 5.9%, 53.0%, and 11.2%, respectively [38]. The discrepancy of these percentages might depend on the characteristics of participants. Therefore, the limitation of the present study is that all participants were recruited at the Okayama University Hospital, and this may limit the ability to extrapolate these findings to the general population.

We used two cut-off points, namely moderate/severe periodontitis and severe periodontitis, because the relationship between coffee consumption and periodontitis severity was unclear. In the two logistic regression models (Table 3 and Table 4), different confounding variables were selected. Effects of these variables on moderate/severe periodontitis and severe periodontitis were different. The effect of nutritional status on moderate periodontitis may be weak because other variables including age and PCR (%) have a strong effect on it. In contrast, although dental plaque can cause periodontitis, PCR (%) may not sufficiently reflect the subgingival plaque and dental calculus in deep periodontal pockets and it may have a weak effect on severe periodontitis. A further study is needed to clarify the relationship between these confounding variables and the periodontitis severity during the maintenance phase of periodontal therapy.

This study has other limitations. We did not record sociological factors, which are known to influence periodontal condition. Also, the reliability and validity of the questionnaire used in our study were not tested sufficiently. The above additional information would increase the validity of the presently established relationship between habitual coffee consumption and severe periodontitis. Additionally, the present study was a cross-sectional study. Further longitudinal studies are needed to clarify the relationship between coffee consumption and periodontitis progression.

## 5. Conclusions

There appears to be an inverse association between drinking ≥1 cup of coffee/day and severe periodontitis during the maintenance phase of periodontal therapy.

## References

[1] Williams R.C. (1990). Periodontal disease. N. Engl. J. Med..

[2] Califano J.V. (2003). Position paper: Periodontal diseases of children and adolescents. J. Periodontol..

[3] Labriola A., Needleman I., Moles D.R. (2005). Systematic review of the effect of smoking on nonsurgical periodontal therapy. Periodontol 2000.

[4] Kinane D.F., Chestnutt I.G. (2000). Smoking and periodontal disease. Crit. Rev. Oral Biol. Med..

[5] Ogawa H., Yoshihara A., Hirotomi T., Ando Y., Miyazaki H. (2002). Risk factors for periodontal disease progression among elderly people. J. Clin. Periodontol..

[6] Lages E.J., Costa F.O., Lages E.M., Cota L.O., Cortelli S.C., Nobre-Franco G.C., Cyrino R.M., Cortelli J.R. (2012). Risk variables in the association between frequency of alcohol consumption and periodontitis. J. Clin. Periodontol..

[7] Salvi G.E., Carollo-Bittel B., Lang N.P. (2008). Effects of diabetes mellitus on periodontal and peri-implant conditions: Update on associations and risks. J. Clin. Periodontol..

[8] D’Aiuto F., Sabbah W., Netuveli G., Donos N., Hingorani A.D., Deanfield J., Tsakos G. (2008). Association of the metabolic syndrome with severe periodontitis in a large U.S. population-based survey. J. Clin. Endocrinol. Metab..

[9] Chapple I.L. (2009). Potential mechanisms underpinning the nutritional modulation of periodontal inflammation. J. Am. Dent. Assoc..

[10] Van der Velden U., Kuzmanova D., Chapple I.L. (2011). Micronutritional approaches to periodontal therapy. J. Clin. Periodontol..

[11] Kushiyama M., Shimazaki Y., Murakami M., Yamashita Y. (2009). Relationship between intake of green tea and periodontal disease. J. Periodontol..

[12] Iwasaki M., Moynihan P., Manz M.C., Taylor G.W., Yoshihara A., Muramatsu K., Watanabe R., Miyazaki H. (2013). Dietary antioxidants and periodontal disease in community-based older Japanese: A 2-year follow-up study. Public Health Nutr..

[13] Adegboye A.R., Christensen L.B., Holm-Pedersen P., Avlund K., Boucher B.J., Heitmann B.L. (2012). Intake of dairy products in relation to periodontitis in older Danish adults. Nutrients.

[14] Lee W.J., Zhu B.T. (2006). Inhibition of DNA methylation by caffeic acid and chlorogenic acid, two common catechol-containing coffee polyphenols. Carcinogenesis.

[15] Oleaga C., Ciudad C.J., Noé V., Izquierdo-Pulido M. (2012). Coffee polyphenols change the expression of STAT5B and ATF-2 modifying cyclin D1 levels in cancer cells. Oxid. Med. Cell Longev..

[16] Gutiérrez-Grobe Y., Chávez-Tapia N., Sánchez-Valle V., Gavilanes-Espinar J.G., Ponciano-Rodríguez G., Uribe M., Méndez-Sánchez N. (2012). High coffee intake is associated with lower grade nonalcoholic fatty liver disease: The role of peripheral antioxidant activity. Ann. Hepatol..

[17] Matsuura H., Mure K., Nishio N., Kitano N., Nagai N., Takeshita T. (2012). Relationship between coffee consumption and prevalence of metabolic syndrome among Japanese civil servants. J. Epidemiol..

[18] Larsson S.C., Wolk A. (2007). Coffee consumption and risk of liver cancer: A meta-analysis. Gastroenterology.

[19] Naganuma T., Kuriyama S., Kakizaki M., Sone T., Nakaya N., Ohmori-Matsuda K., Nishino Y., Fukao A., Tsuji I. (2008). Coffee consumption and the risk of oral, pharyngeal, and esophageal cancers in Japan: The Miyagi Cohort Study. Am. J. Epidemiol..

[20] Lopez-Garcia E., van Dam R.M., Qi L., Hu F.B. (2006). Coffee consumption and markers of inflammation and endothelial dysfunction in healthy and diabetic women. Am. J. Clin. Nutr..

[21] Ng N., Kaye E.K., Garcia R.I. (2014). Coffee consumption and periodontal disease in men. J. Periodontol..

[22] Brown L.J., Oliver R.C., Löe H. (1989). Periodontal diseases in the U.S. in 1981: Prevalence, severity, extent, and role in tooth mortality. J. Periodontol..

[23] O’Leary T.J., Drake R.B., Naylor J.E. (1972). The plaque control record. J. Periodontol..

[24] Naganuma T., Kuriyama S., Akhter M., Kakizaki M., Nakaya N., Matsuda-Ohmori K., Shimazu T., Fukao A., Tsuji I. (2007). Coffee consumption and the risk of colorectal cancer: A prospective cohort study in Japan. Int. J. Cancer.

[25] Kokubo Y., Iso H., Saito I., Yamagishi K., Yatsuya H., Ishihara J., Inoue M., Tsugane S. (2013). The impact of green tea and coffee consumption on the reduced risk of stroke incidence in Japanese population: The Japan public health center-based study cohort. Stroke.

[26] Eke P.I., Page R.C., Wei L., Thornton-Evans G., Genco R.J. (2012). Update of the case definitions for population-based surveillance of periodontitis. J. Periodontol..

[27] Tomofuji T., Furuta M., Ekuni D., Irie K., Azuma T., Iwasaki Y., Morita M. (2011). Relationships between eating habits and periodontal condition in university students. J. Periodontol..

[28] Persson G.R., Hitti J., Paul K., Hirschi R., Weibel M., Rothen M., Persson R.E. (2008). *Tannerella forsythia* and *Pseudomonas aeruginosa* in subgingival bacterial samples from parous women. J. Periodontol..

[29] Takeda Y., Horii N., Mituzaki J., Tanaka H., Andoh Y., Suzuki M., Miyashita H. (1990). Improvement of plaque score after oral hygiene instruction in patients with periodontitis. Nihon Shishubyo Gakkai Kaishi.

[30] Acharya A., VanWormer J.J., Waring S.C., Miller A.W., Fuehrer J.T., Nycz G.R. (2013). Regional epidemiologic assessment of prevalent periodontitis using an electronic health record system. Am. J. Epidemiol..

[31] Rivas-Tumanyan S., Campos M., Zevallos J.C., Joshipura K.J. (2013). Periodontal disease, hypertension, and blood pressure among older adults in Puerto Rico. J. Periodontol..

[32] Saffi M.A., Furtado M.V., Montenegro M.M., Ribeiro I.W., Kampits C., Rabelo-Silva E.R., Polanczyk C.A., Rösing C.K., Haas A.N. (2013). The effect of periodontal therapy on C-reactive protein, endothelial function, lipids and proinflammatory biomarkers in patients with stable coronary artery disease: Study protocol for a randomized controlled trial. Trials.

[33] Maldonado G., Greenland S. (1993). Simulation study of confounder-selection strategies. Am. J. Epidemiol..

[34] Huang J., de Paulis T., May J.M. (2004). Antioxidant effects of dihydrocaffeic acid in human EA.hy926 endothelial cells. J. Nutr. Biochem..

[35] Tamaki N., Tomofuji T., Maruyama T., Ekuni D., Yamanaka R., Takeuchi N., Yamamoto T. (2008). Relationship between periodontal condition and plasma reactive oxygen metabolites in patients in the maintenance phase of periodontal treatment. J. Periodontol..

[36] Pham N.M., Wang Z., Morita M., Ohnaka K., Adachi M., Kawate H., Takayanagi R., Kono S. (2011). Combined effects of coffee consumption and serum γ-glutamyltransferase on serum C-reactive protein in middle-aged and elderly Japanese men and women. Clin. Chem. Lab. Med..

[37] Yamashita K., Yatsuya H., Muramatsu T., Toyoshima H., Murohara T., Tamakoshi K. (2012). Association of coffee consumption with serum adiponectin, leptin, inflammation and metabolic markers in Japanese workers: A cross-sectional study. Nutr. Diabetes.

[38] Thornton-Evans G., Eke P., Wei L., Palmer A., Moeti R., Hutchins S., Borrell L.N. (2013). Periodontitis among adults aged ≥30 years—United States, 2009–2010. MMWR.

